# Contributions of intermittently scanned continuous glucose monitoring frequency and bolus insulin dosing on time in range: Analysis of data from CGM and connected insulin pens

**DOI:** 10.1111/dom.70165

**Published:** 2025-10-03

**Authors:** Pratik Choudhary, Kalvin Kao, Farhan Quadri, Elemer Balogh, Jody Foster

**Affiliations:** ^1^ Diabetes Research Centre University of Leicester Leicester UK; ^2^ Abbott Diabetes Care Alameda California USA; ^3^ Abbott Diabetes Care Maidenhead UK

**Keywords:** CGM, connected insulin pen, glycaemic variability. Interconnected devices, time above range, time in range

## Abstract

**Background and Aims:**

Integration of data from continuous glucose monitoring (CGM) and connected insulin pens allows us to investigate their use to optimise glucose control. This study examines how insulin bolus frequency reported by connected pens and frequency of glucose scans by an intermittently‐scanned continuous glucose monitoring (isCGM) system relate to glycaemic control in a population of real‐world European users.

**Methods:**

Data from glucose sensors and connected insulin pens were aggregated for LibreView users who integrated their connected pen by January 1, 2024. The most recent 90‐day window with ≥30 days' glucose data and ≥15 days of insulin bolus doses following their integration date was analysed. We stratified users by categories of average isCGM scan frequency: low (<6.1 scans/day), medium (6.1–14.0 scans/day) or high (>14.0 scans/day), and average bolus frequency: low (<3.1 boluses/day), medium (3.1–6.7 boluses/day) or high (>6.7 boluses/day).

**Results:**

Data from 10,993 users was available over 80.6 days/user. Median scans/day were 9.3 [6.1–14.0] and median bolus/day was 4.5 [3.1–6.7]. Increased daily scans were associated with greater time in range (TIR) 70–180 mg/dL (3.9–10.0 mmol/L) within each of the low, medium, and high bolus frequency groups. Increased bolus frequency was associated with increased TIR in the lowest scanning frequency group. Similar outcomes were observed for time above range (TAR) and glycaemic variability.

**Conclusions:**

While glucose monitoring frequency and insulin bolus dosing are both indicators of engagement with diabetes self‐management, higher TIR has a closer association with increased rates of user engagement with isCGM than with increased rates of bolus dosing.

## 1 INTRODUCTION

The introduction of continuous glucose monitoring (CGM) systems for the care of people with either type 1 diabetes (T1D) or type 2 diabetes (T2D) treated with insulin has delivered significant improvements in glycaemia, with lower levels of glycated haemoglobin (HbA1c),^1^, ^2^ reduced hypoglycaemia,^3^, ^4^ along with improvements in patient‐reported distress and quality of life.^5^, ^6^ Data from a number of large retrospective studies have shown a correlation between frequency of scans or views with glucose outcomes.^7^, ^8^, ^9^ Estimated HbA1c gradually reduced from 8.0% to 6.7% (64 to 50 mmol/mol) as scan rate increased from lowest to highest scan groups (4.4 and 48.1 scans/day, respectively; *p* < 0.001), and time below 3.9, 3.1, and 2.5 mmol/L decreased by 15%, 40%, and 49%, respectively (all *p* < 0.001).

Despite increasing use of continuous subcutaneous insulin infusion (CSII) pumps, the majority of people with T1D remain on multiple daily injections (MDI) with insulin,^10^ and MDI therapy is used for glycaemic control for significant numbers of people with T2D.^11^ For people with diabetes on insulin, optimal glycaemic management can be hindered by missed or mistimed insulin doses.^12^

Connected insulin pens capture and report information on insulin doses and timings, with the goal of helping the person with diabetes to optimise treatment outcomes.^13^, ^14^ Many insulin pens can be connected with CGM systems to further improve insulin therapy by supporting adherence with MDI, with enhanced safety by minimising hypoglycaemia.^15^ Small‐scale studies have shown that introducing connected insulin pens alongside CGM for children with T1D (*n* = 39) results in significantly reduced time below range (TBR) with Level 2 hypoglycaemia <54 mg/dL (<3.0 mmol/L), although missed mealtime boluses were not impacted.^16^ In adults with T1D (*n* = 32), introducing Novopen 6 connected pens revealed that the estimated probability of missing at least one basal‐insulin dose over a 14‐day period was 22%,^17^ and that missed basal doses were associated with significantly increased mean glucose, reduced time in range (TIR) 70–180 mg/dL (3.9–10.0 mmol/L) and higher glucose management indicator (GMI). A retrospective observational study on adults with T1D (*n* = 1194) has shown that the number of daily bolus injections is positively associated with increased TIR,^18^ and that individuals delivering three daily bolus insulin injections on average had an estimated 11% chance of achieving >70% TIR. Connected insulin pen engagement, as measured by the number of days with connected insulin pen data uploads over the previous 14 days, was also significantly associated with increased TIR. A larger retrospective study of 3945 adults with diabetes (diabetes type not recorded for 81.6% of participants)^19^ also showed that the number of daily bolus injections is correlated with TIR over a 14‐day period, and calculated that a single missed basal‐insulin dose over a 14‐day period was associated with a −2.8% decrease in TIR and a 0.2% increase in GMI, and a single missed basal‐insulin dose reduced TIR by −1.7% and increased GMI by 0.1%. This analysis also found that, over a 14‐day period, an average of 6 bolus doses were missed.

To date, limited analysis has been conducted to understand how insulin bolus frequency and glucose monitoring frequency interact to support glycaemic management for people with diabetes on MDI therapy. In November 2022, the Novopen 6 connected insulin pen was integrated with the LibreView CGM data platform, allowing insulin dosing data to be recorded alongside glucose metrics captured by an intermittently scanned CGM system (isCGM) (FreeStyle Libre or FreeStyle Libre 2, Abbott Diabetes Care, Alameda, CA). The aim of the study reported here was to examine the relative impacts of isCGM engagement and prandial insulin engagement for isCGM users in Europe.

## 2 MATERIALS AND METHODS

### 2.1 Glucose and insulin bolus data

**FIGURE 1:**
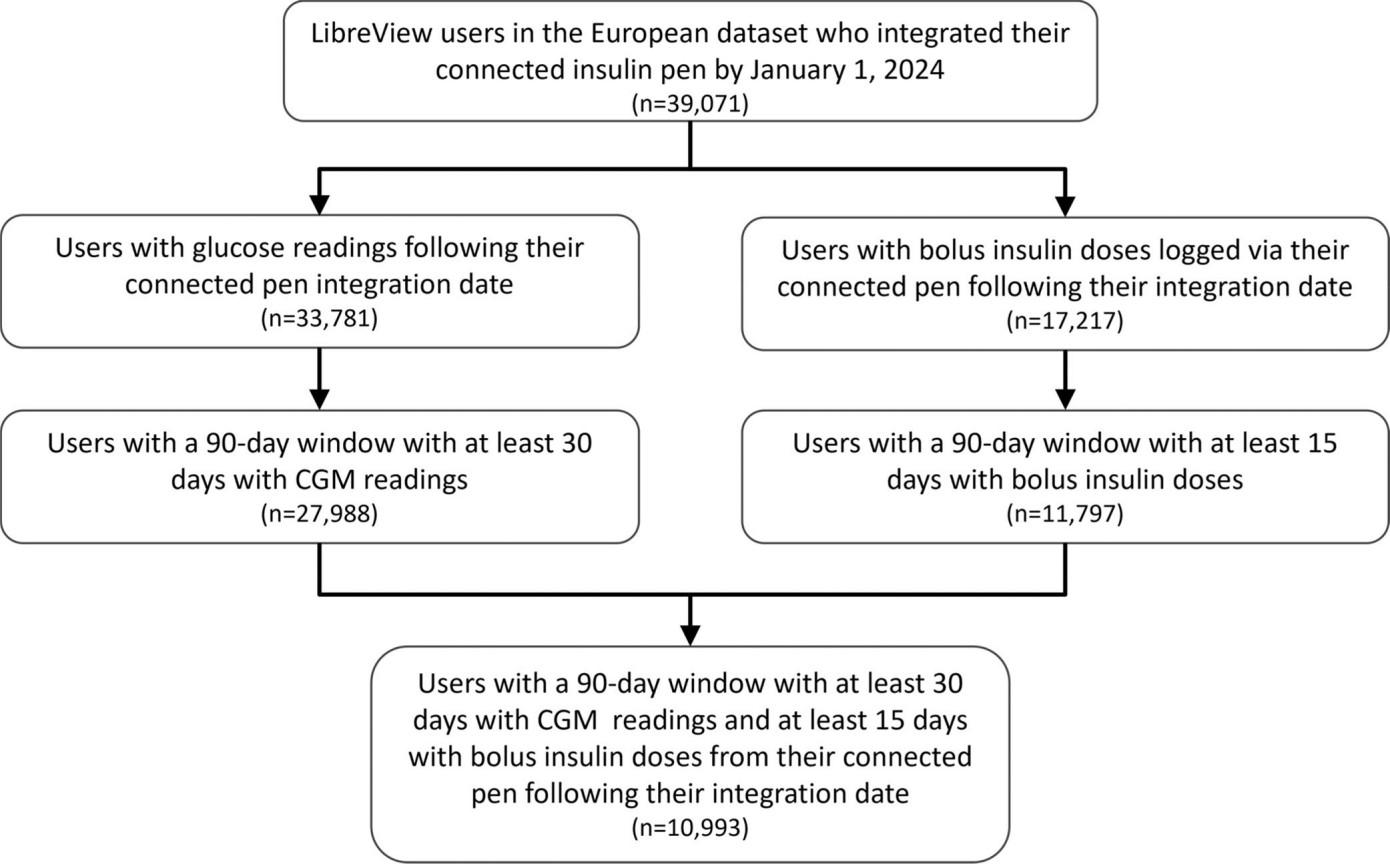
Selection algorithm for LibreView users with glucose data and associated connected insulin pen bolus insulin data. Algorithm describes identification of LibreView users' combined CGM and connected insulin pen data that is representative of their typical behaviour when using both in tandem. CGM, continuous glucose monitoring.

Glucose data from two isCGM sensor types (FreeStyle Libre and FreeStyle Libre 2) is stored in the LibreView online database that can be accessed and viewed by users and shared with their healthcare professionals. FreeStyle Libre users are required to scan their sensor to get a real‐time glucose measurement, whereas FreeStyle Libre 2 users have glucose readings streamed to their app or reader in real‐time. Users of both sensor types are encouraged to scan their sensor in order to collect and backfill historical glucose readings that can be uploaded to LibreView. During consenting, LibreView users can opt in to their glucose and product‐related data being de‐identified and aggregated for research purposes. isCGM glucose data and associated Novopen 6 bolus‐insulin data were collected from LibreView users in the EMEA region (which included a small number of users from the Middle East and Africa) who integrated their connected insulin pen by January 1, 2024. Users' 90‐day window with the highest data capture (defined as the proportion of days with at least one logged bolus dose) following their integration date were considered for analysis, and amongst these, only periods with at least 30 days with glucose readings and at least 15 days with bolus doses were selected for final analysis. These selection choices ensure that: (1) our analysis sample contains the most complete examples available of simultaneous engagement with CGM and integrated pen data, and that; (2) users' sampled data is indeed representative of their typical engagement behaviours and glucose control. In the case of a user having multiple such eligible windows, only their most recent was selected for analysis. This selection criteria is visualised in Figure 1. Age‐group categories of selected users, based on self‐reported age in LibreView accounts (0–5, 6–12, 13–17, 18–25, 26–49, 50–64, 65+ years), were also collected and reported in order to contextualise the sample's observed engagement behaviours and glucose outcomes.

### 2.2 User engagement stratification

CGM engagement was based on quartiles of users' mean daily scan rates and categorised as low (<6.1 scans/day), medium (6.1–14.0 scans/day), or high (>14 scans/day), representing <25th, 25–75th, and >75th centiles. Similarly, insulin bolus engagement was defined as low (<3.1 boluses/day), medium (3.1–6.7 boluses/day), or high (>6.7 boluses/day), based on <25th, 25–75th, and >75th centiles. The glycaemic metrics assessed for each of the resulting nine combinations of monitoring and bolus frequency levels were: glycaemic variability, as measured by mean standard deviation (SD), mean %TIR, mean %time above range (TAR) >180 mg/dL (>10.0 mmol/L), and mean %TAR >250 mg/dL (>13.9 mmol/L), as well as median %TBR <54 mg/dL (<3.0 mmol/L) and median %TBR <70 mg/dL (<3.9 mmol/L), due to the skew in the TBR distributions.

### 2.3 Comparative Sample

We also selected a separate sample of LibreView users in the EU for comparison– those with at least 30 days of CGM readings following March 1, 2024, and no integrated insulin pen data (Figure S1). Each user's TIR was calculated to produce a cumulative frequency distribution of TIR representing the greater population of LibreView users, in order to contextualise the glucose control of the selected sample of connected insulin pen users that is the main subject of investigation.

### 2.4 Statistical analysis

The database was analysed by structured query language routines and the Python programming language (www.python.org), and hypothesis tests for a difference in correlation coefficients were conducted with the R programming language (http://www.r-project.org), specifically the *cocor* package (https://cran.r-project.org/web/packages/cocor/).

## 3 RESULTS

**FIGURE 2:**
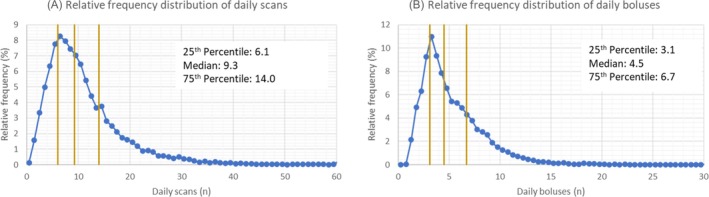
Frequency distributions of daily scans and daily boluses for LibreView users with connected insulin pen data. Figure 2A shows the relative frequency of analysed users in each scan rate bin. The plot shows 60 bins, with each bin size being 1 daily scan. Vertical lines indicate the 25th, 50th, and 75th percentiles of daily scans. Figure 2B shows the relative frequency of analysed users in each bolus rate bin. The plot shows 60 bins, with each bin size being a 0.5 daily bolus. Vertical lines indicate the 25th, 50th, and 75th percentiles of daily boluses.

The European LibreView dataset yielded 39,071 isCGM users who integrated their connected insulin pen by January 1, 2024. Amongst this group, we identified 10,993 LibreView users (28.1% of the total population identified) who met the selection criteria and thereby contributed at least 30 days of CGM data and at least 15 days of connected insulin pen bolus doses (Figure 1) to this analysis. The age demographic composition of these users is shown in Table 1, and the International distribution is shown in Table 2. The majority of individuals were aged 18+ years (92.1%) and the largest national user groups represented were from Spain (39.8%) and the United Kingdom (30.6%). The distribution of daily scan rates and insulin bolus rates is shown in Figure 2, along with the interquartile ranges used to create the different categories. The frequency distribution of users amongst the nine segments is shown in Table S1. The 25th, 50th, and 75th centile of TIR was similar between the cohort (*n* = 10,993) using connected pens and the comparator cohort (*n* = 669,305) not using connected pens (Figure S1).

**TABLE 1:** LibreView users with connected insulin pen data by age group.

Age group (years)	Number of users	Proportion (%)
0–5	21	0.2
6–12	287	2.6
13–17	568	5.2
18–25	995	9.1
26–49	5108	46.6
50–64	2925	26.7
65+	1065	9.7

**TABLE 2:** LibreView users with connected insulin pen data by national location.

Country	Number of users	Proportion (%)
Spain	4417	39.8
United Kingdom	3399	30.6
Belgium	637	5.7
Poland	482	4.3
Denmark	437	3.9
Finland	417	3.8
Czech Republic	413	3.7
Sweden	332	3.0
Portugal	251	2.3
Netherlands	94	0.8
Italy	81	0.7
Greece	54	0.5
Ireland	24	0.2
Slovenia	22	0.2
Lithuania	19	0.2
Hungary	17	0.2
South Africa	2	0.0
Turkey	1	0.0

### 3.1 Time in range for LibreView users with a connected insulin pen

**FIGURE 3:**
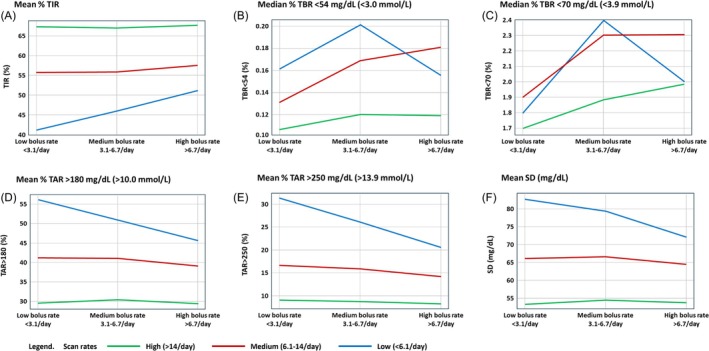
CGM metrics of glycaemia for LibreView users, segmented by scan rate and insulin bolus frequency. SD, standard deviation; TAR, time above range; TBR, time below range; TIR, time in range.

The interaction between daily scanning behaviour and insulin bolus frequency for TIR is shown in Figure 3A and Table S2a. For LibreView users with low scanning rates (<6 scans/day), those with the fewest daily boluses had a mean TIR of 41.1%, contrasting with a mean TIR of 51.1% for those with the highest daily bolus frequency. We observe a similar pattern, to a lesser degree, in the group of users with medium scanning rates (6.1–14 scans/day)—those with <3.1 daily boluses on average had a mean TIR of 55.7%, and those with >6.7 boluses/day had a mean TIR of 57.7%. For the highest scan frequency group with >14.0 scans/day, the mean TIR was similar between those with low (67.4%) and high (67.7%) bolus frequency.

Amongst users with the fewest daily boluses, the 41.1% mean TIR for those with the lowest scan frequency may also be contrasted with the 67.4% mean TIR for those with the highest scan frequency. For users with 3.1–6.7 daily boluses on average, we observe a 45.9% mean TIR when mean daily scans are <6.1% and 67.1% when daily scans are >14. In the group with the highest daily bolus frequency, mean TIR is 51.1% when daily scans are <6.1 and is again >67% when daily scans are high.

**FIGURE 4:**
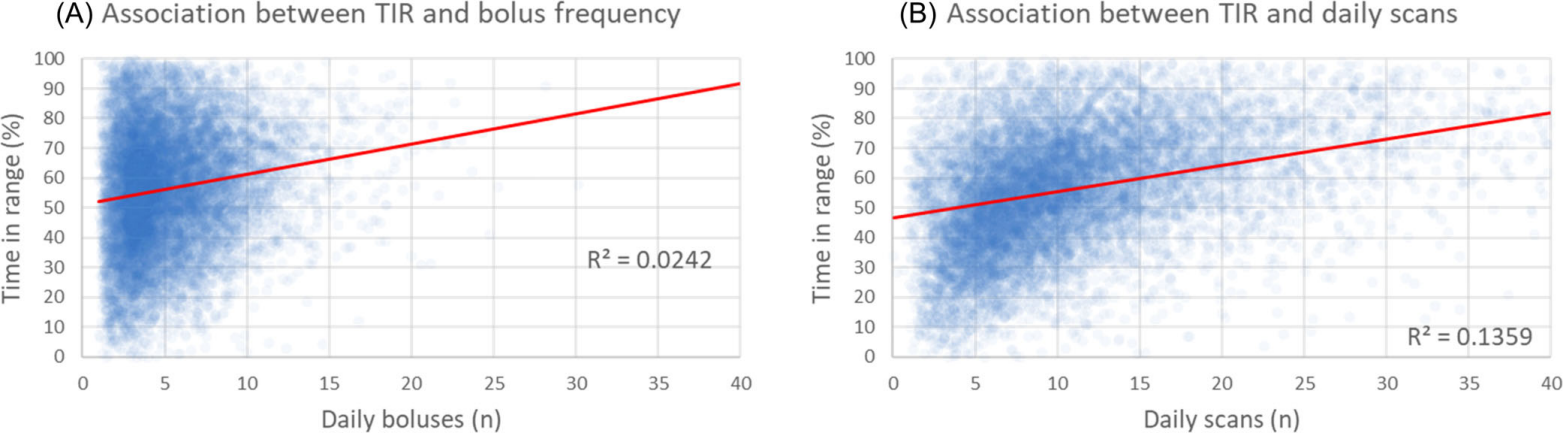
Correlations between bolus frequency, scan frequency, and time in range for LibreView users with integrated connected insulin pen data. Figure 4A shows isCGM users' bolus frequency and TIR data. The Spearman correlation coefficient for this association = 0.15. Darker areas indicate a greater occurrence of observations. Figure 4B shows isCGM users' scan frequency and TIR data. The Spearman correlation coefficient for this association = 0.44. Darker areas indicate a greater occurrence of observations. Plot excludes outlier scan frequencies above 40 scans/day.

The associations between TIR and bolus frequency, and between TIR and isCGM scan frequency are shown in Figure 4, with Spearman Correlation Coefficients of 0.15 and 0.44, respectively. A Fisher's Z transformation on those coefficients, followed by an Olkin's Z statistic, for the TIR‐daily bolus association and the TIR‐daily scan association was highly significant (*p* < 0.0001) and confirmed that the strength of association greatly favours the influence of isCGM daily scan rates over daily bolus frequencies. Observations from a subgroup analysis confirm that this relationship between increased isCGM daily scans and improved TIR holds true for each of the separate age groups in the analysis set, including paediatrics (data not shown).

### 3.2 Time below range for LibreView users with a connected insulin pen

Median %TBR <54 mg/dL (<3.0 mmol/L) and %TBR <70 mg/dL (<3.9 mmol/L) appeared to be generally low for all analysis groups, regardless of the scan frequency or daily bolus insulin dose rates (Figure 3B,C, Table S2b,c).

### 3.3 Time above range for LibreView users with a connected insulin pen

Mean TAR follows a similar pattern to that of TIR (Figure 3D,E, Table S2d,e). isCGM users with low daily scanning rates demonstrated a mean TAR >180 mg/dL (>10.0 mmol/L) of 56.0% when daily boluses were fewer than 3.1 and 45.8% when daily boluses were greater than 6.7. When mean daily scans were greater than 14, we instead observe a mean TAR >180 mg/dL (>10.0 mmol/L) of 29.7% and 29.3% when daily boluses were <3.1 and >6.7, respectively (Figure 3D, Table S2d).

Observations of TAR >250 mg/dL (>13.9 mmol/L) mirror those of TAR >180 mg/dL (>10.0 mmol/L). isCGM users with low daily scanning rates demonstrated a mean TAR >250 mg/dL (>13.9 mmol/L) of 31.2% when daily boluses were less than 3.1% and 20.4% when daily boluses were greater than 6.7. However, when mean daily scans were greater than 14, we observe that the mean TAR >250 mg/dL (>13.9 mmol/L) was 9.1% and 8.2% for those who had <3.1 daily boluses and >6.7 daily boluses, respectively.

### 3.4 Glycaemic variability for LibreView users with a connected insulin pen

The SD levels for isCGM users with low daily scanning rates were 82.7 mg/dL (4.6 mmol/L) for those with less than 3.1 daily boluses and 71.1 mg/dL (3.9 mmol/L) for those with greater than 6.7 daily boluses (Figure 3F). For users with high daily scan rates, mean glucose SD was 53.4 mg/dL (3.0 mmol/L) and 53.9 mg/dL (3.0 mmol/L) when daily boluses were <3.1 and >6.7, respectively (Figure 3F, Table S2f).

## 4 DISCUSSION

The outcomes of this analysis of a large European population of LibreView users with integrated connected insulin pen bolus data reveal that daily scan rates have a stronger association with time in range (TIR) than bolus frequency does. The correlation coefficient of 0.44 indicates a moderate positive association between TIR and isCGM scan frequency, while the coefficient of 0.15 reflects only a weak positive association between TIR and bolus frequency. This association is more apparent for the group of users with the lowest bolus frequency (<3.1 boluses/day), whose TIR is 67% in those with >14 scans compared to 41.1% if <6.1 scans/day. Interestingly, while we observe a TIR of 67% in those with low bolus frequency but high scan frequency, the TIR achieved in those with high bolus frequency (>6.7 boluses/day) but low scanning frequency is only 51.1%.

In this large sample of 10,993 LibreView users with integrated connected insulin pen data, rates of level 1 < 70 mg/dL (<3.9 mmol/L) and level 2 < 54 mg/dL (<3.0 mmol/L) hypoglycaemia were reassuringly low, with %TBR well below international consensus recommendations for <4% and <1% TBR, respectively.^20^ That our data do not show an impact on hypoglycaemia for isCGM users in conjunction with connected pens is not at odds with other studies. One study analysing data from 1194 adults with T1D using connected insulin pens and CGM reported a significant increase in TBR <70 mg/dL (<3.9 mmol/L) with increasing bolus frequency,^18^ as well as significantly increased TIR 70–180 mg/dL (3.9–10.0 mmol/L) and reduced glycaemic variability. In contrast, a smaller study (*n* = 94) in T1D pairing CGM and connected insulin pen data found small reductions in level 1 TBR 54–69 mg/dL (3.0–3.8 mmol/L) after initiation of connected insulin pen therapy, and a significant reduction in level 2 TBR <54 mg/dL (<3.0 mmol/L).^21^ A 2025 study using de‐identified data from LibreView, Glooko, or Diasend also found increased TIR for people using CGM in the 12 months after starting connected insulin pen therapy but no increase in TBR, rather showing a small decrease.^22^ In our own study, using de‐identified data, we are not able to assess the impact of the interaction of the study cohort with alarms that prevent them from going below 4 mmol/L, and we do not have a measure of how often the users took carbohydrate or felt symptomatic. Thus, it is clear that further studies are warranted to dissect the impact of CGM use, insulin smart pens, and measures of hypoglycaemia.

These data show that increased engagement with both bolusing and scanning is associated with reduced time in the very highest glucose readings as expected and this may have clinical value in reducing complications as well as the risk of DKA and hyperglycaemia‐related complications.

At the lowest scan frequency, mean %TAR >250 mg/dL (>13.9 mmol/L) was 31.2%, 26.0%, and 20.4% when daily boluses were <3.1, 3.1–6.7, and >6.7, respectively, showing that increased boluses are associated with increased TIR. However, in those with >14 scans/day, the association between bolus frequency and TAR >180 (>10.0 mmol/L) or >250 mg/dL (>13.9 mmol/L) is unclear. This may reflect a cohort of patients with high anxiety around diabetes or hypoglycaemia, where the high scanning rate reflects the higher anxiety, but an increased number of boluses is not able to reduce glucose. In clinical practice, we can also see patients whose education and therapy are not optimised, so they have high variability with glucose swinging from high to low despite the greater number of boluses.

A further finding from our study is that higher scanning frequency is associated with lower glycaemic variability, rather than with more frequent insulin bolusing. For isCGM users with either low, medium, or high bolus frequencies, glycaemic variability as measured by SD was on average 82.7 mg/dL (4.6 mmol/L), 79.3 mg/dL (4.4 mmol/L), and 72.1 mg/dL (4.0 mmol/L), respectively, when daily scans were low, and 53.4 mg/dL (3.0 mmol/L), 54.5 mg/dL (3.0 mmol/L), and 53.9 mg/dL (3.0 mmol/L), respectively, when daily scans were high. This suggests a high degree of engagement with glucose values may affect behaviours that reduce glucose variability, such as the timing of rapid acting insulin.

Our data do support previous retrospective studies that have reported improved TIR, alongside reduced TAR (both >180 mg/dL [>10.0 mmol/L] and >250 mg/dL [>13.9 mmol/L]) and lower glycaemic variability with increased daily insulin bolus frequencies for adult connected insulin pen users with T1D^18^ and for people with diabetes on basal‐bolus insulin therapy, but diabetes type not known.^19^ Prospective studies have also shown that people with diabetes using connected insulin pens and able to view their CGM have increased TIR and reduced TAR compared to users without access to CGM readings.^15^, ^21^ A prospective observational study of 94 adults with T1D showed that using connected insulin pens by CGM users resulted in clinically meaningful increases in TIR (8.5%) and corresponding reductions in TAR, associated with a 43% reduction in missed mealtime insulin boluses.^21^ This study also reported significant reductions in glycaemic variability and level 2 hypoglycaemia (<54 mg/dL). Increased TIR has also been reported using CGM and insulin bolusing data from LibreView, Glooko, or Diasend.^22^ Overall, these studies have highlighted the value of connected insulin pens to confirm that insulin boluses are administered, as well as allowing the person with diabetes and their healthcare provider to regularly review and adjust their insulin dosing and/or food intake appropriately. Our outcomes for the group of LibreView users with integrated connected insulin pen data, but in the lowest 25th percentile of daily scan rates, clearly show that increased bolus dosing is associated with increased TIR, as well as decreased TAR >180 mg/dL (>10.0 mmol/L) and TAR >250 mg/dL (>13.9 mmol/L). All of these observations make sense since adherence to daily insulin therapy is well documented to improve glycaemia for people with diabetes treated with insulin, either as basal or bolus doses.^17^, ^21^, ^23^ The positive impact of using connected insulin pens adds to the options for maintaining and further improving treatment engagement and adherence. It is also accepted that connected insulin pens and the data extracted from them have limitations, and that future development will increase their role in technology‐driven models of diabetes care.^24^

The notable finding from our study is that increased engagement with CGM for people with diabetes on insulin therapy can be more impactful than more‐frequent daily insulin bolus dosing. Indeed, combined analysis of TIR, TAR, and TBR outcomes of our study shows that, at all insulin engagement levels, increased CGM engagement is associated with increased TIR and decreased TAR (Figures 3 and 4), and that this relationship occurs for connected insulin pen users without an associated increase in time in hypoglycaemia (Figure 3). Furthermore, those with the greatest CGM engagement exhibit a high level of glucose control, and this may reflect evidence of behaviour modification as a result of CGM data rather than increases in insulin dosing. Our data do not provide any insights into what other decisions LibreView users are making or what actions are prompted by their use of the isCGM system. However, we can hypothesise that what they learn from their glucose reading and the associated trend arrows, prompts a decision that has positive impact on their TIR and TAR. These may be related to (but not limited to) snacking or mealtime behaviours (e.g., better timing of insulin boluses), or physical activity. In this context, the act of intermittently scanning a CGM sensor has allowed us to track the user behaviours associated with viewing and assessing their glucose levels. For users of real‐time CGM devices, this scanning event is not required and thus it is not possible to objectively evaluate this act of engagement. However, we would hypothesise that for real‐time CGM users the act of simply viewing their glucose readings has the same consequences for decision‐making that can impact their TIR and TAR, as it does for isCGM users who scan and view their glucose readings.

## 5 STRENGTHS AND LIMITATIONS

A key strength of this analysis is the largest cohort of individuals with diabetes using CGM in conjunction with connected insulin pens, in which we have been able to examine both the impact of isCGM engagement and insulin bolus frequency, which has not been possible with other studies. The international complexion of the cohort is another strength. A clear limitation is that our study includes only de‐identified data, with no information regarding patient behaviours or motivations in regard to either isCGM or connected insulin pens, which is a significant restriction. Also, the nations represented are predominantly white European or Hispanic, with limited other ethnic representation, which limits the generalisability of the findings. Another limitation is that the data are anonymised and do not allow us to identify subgroups with either T1D or T2D and to investigate any distinct glucose monitoring or insulin bolusing profiles that may characterise each of these subgroups. Another potential limitation is the need for connected insulin pen users to actively transfer the insulin bolus data to the LibreView app/reader. This may mean that some boluses are not added to the system, particularly for new connected pen users, resulting in more complete CGM data compared to insulin bolus data. We must also acknowledge that some insulin boluses may have been delivered using standard non‐connected insulin pens available to the users. No data on basal‐insulin dosing was available or included in our study, which is a limitation. Based on the time of the data collection in relation to the national reimbursement programmes for the use of isCGM sensors, we assume that the large majority of the users were persons with T1D, although this is not confirmed by our data. However, these limitations may also be mitigated by our observations (Figure S1), which demonstrate via cumulative frequency distributions of TIR that this sample under study has similar glucose control as the greater LibreView population, thereby increasing the generalisability of our results and analyses.

## 6 CONCLUSIONS

This novel analysis has examined the interaction between CGM monitoring frequencies and insulin bolus frequencies with regard to glucose control, for a large cohort of LibreView users with integrated connected insulin pen data. Our analysis shows that increased engagement with the isCGM system for people with diabetes has a greater association with glycaemic control than does more‐frequent insulin bolus dosing. We observed that favourable TIR, TAR, and glycaemic variability in our study cohort were consistently achieved by LibreView users with higher scanning frequencies whether they demonstrated low, medium, or high engagement with insulin bolus dosing. In contrast, the associations between increased TIR, decreased TAR, decreased glycaemic variability, and increased bolus insulin dosing frequencies were only evident in the group of LibreView users with the lowest daily scanning behaviours. We believe that these outcomes speak to the value of CGM‐derived biofeedback for the person with diabetes in making daily diabetes self‐management decisions, even when operating within a digitally interconnected self‐care environment.

## AUTHOR CONTRIBUTIONS

All named authors contributed to the concept and design of the manuscript and worked collaboratively to review and prepare the final manuscript.

## FUNDING INFORMATION

Funding for this study was provided by Abbott Diabetes Care.

## CONFLICT OF INTEREST STATEMENT

PC has received personal fees from Abbott Diabetes Care, Dexcom, Diasend, Eli Lilly, Insulet, Medtronic, Novo Nordisk, Roche, Sanofi Aventis. KK, FQ, EB and JF are employees of Abbott Diabetes Care.

## PEER REVIEW

The peer review history for this article is available at https://www.webofscience.com/api/gateway/wos/peer‐review/10.1111/dom.70165.

## Supporting information


**Table S1.** Proportion of LibreView users with integrated connected insulin pen data segmented by scan rate and bolus frequency.
**Table S2.** CGM metrics of glycaemia for LibreView users, segmented by scan rate and insulin bolus frequency.
**Figure S1.** Comparison of relative frequency of TIR between users of an integrated connected insulin pen and other LibreView users in Europe.

## Data Availability

The data that support the findings of this study are available from the corresponding author upon reasonable request.
