# Supplementary material for: Contributions of intermittently scanned continuous glucose monitoring frequency and bolus insulin dosing on time in range: Analysis of data from CGM and connected insulin pens
Source: Diabetes Obes Metab. 2025 Oct 3;27(12):7570–7. doi: 10.1111/dom.70165 (PMC12587231; doi:10.1111/dom.70165)
Supplement: Supplementary file 1 — Table S1. Proportion of LibreView users with integrated connected insulin pen data segmented by scan rate and bolus frequency. Table S2. CGM metrics of glycaemia for LibreView users, segmented by scan rate and insulin bolus frequency. Figure S1. Comparison of relative frequency of TIR between users of an integrated connected insulin pen and other LibreView users in Europe. [file DOM-27-7570-s001.pdf]

Contributions of intermittently scanned continuous glucose monitoring frequency and bolus insulin dosing on time in range: analysis of data from CGM and connected insulin pens

Pratik Choudhary,[1] Kalvin Kao,[2] Farhan Quadri,[2] Elemer Balogh,[3] Jody Foster[3]

1. Diabetes Research Centre, University of Leicester, Leicester, UK

2. Abbott Diabetes Care, Alameda, California, USA

3. Abbott Diabetes Care, Maidenhead, UK

Corresponding author: Pratik Choudhary, email Pratik.choudhary@leicester.ac.uk

Supplementary materials

Supplementary Table 1. Proportion of LibreView users with integrated connected insulin pen data segmented by scan rate and bolus frequency

	Daily scan rate/day, n (% of total users)			
**Boluses/day n (% of total users)**	**<6.1**	**6.1-14.0**	**>14.0**	**Total**
**<3.1**	940 (8.6)	1,318 (12.0)	443 (4.0)	2,701 (24.6)
**3.1-6.7**	1,310 (11.9)	2,865 (26.1)	1,341 (12.2)	5,516 (50.2)
**>6.7**	502 (4.6)	1,291 (11.7)	983 (8.9)	2,776 (25.2)
**Total**	2,752 (25.0)	5,474 (49.8)	2,767 (25.2)	10,993 (100)

The numbers and proportions of LibreView users in each high and low frequency category reflect that the 25th and 75th percentiles of those measures were used to define the high and low frequency rates, with the medium category being defined as between these boundaries.

Supplementary Table 2. CGM metrics of glycaemia for LibreView users, segmented by scan rate and insulin bolus frequency

(a) Mean %TIR

	Daily isCGM scan rate/day		
**Boluses/day**	**<6.1**	**6.1-14.0**	**>14.0**
**<3.1**	41.1%	55.7%	67.4%
**3.1-6.7**	45.9%	55.9%	67.1%
**>6.7**	51.1%	57.7%	67.7%

(b) Median %TBR <54 mg/dL (<3.0 mmol/L)

	Daily isCGM scan rate/day		
**Boluses/day**	**<6.1**	**6.1-14.0**	**>14.0**
**<3.1**	0.16%	0.13%	0.11%
**3.1-6.7**	0.20%	0.17%	0.12%
**>6.7**	0.16%	0.18%	0.12%

(c) Median %TBR <70 mg/dL (<3.9 mmol/L)

	Daily isCGM scan rate/day		
**Boluses/day**	**<6.1**	**6.1-14.0**	**>14.0**
**<3.1**	1.8%	1.9%	1.7%
**3.1-6.7**	2.4%	2.3%	1.9%
**>6.7**	2.0%	2.3%	2.0%

(d) Mean %TAR >180 mg/dL (>10.0 mmol/L)

	Daily isCGM scan rate/day		
**Boluses/day**	**<6.1**	**6.1-14.0**	**>14.0**
**<3.1**	56.0%	41.3%	29.7%
**3.1-6.7**	50.9%	40.9%	30.1%
**>6.7**	45.8%	39.1%	29.3%

(e) Mean %TAR >250 mg/dL (>13.8 mmol/L)

	Daily isCGM scan rate/day		
**Boluses/day**	**<6.1**	**6.1-14.0**	**>14.0**
**<3.1**	31.2%	16.7%	9.1%
**3.1-6.7**	26.0%	15.9%	8.7%
**>6.7**	20.4%	14.3%	8.2%

(f) Mean SD (mg/dL)

	Daily isCGM scan rate/day		
**Boluses/day**	**<6.1**	**6.1-14.0**	**>14.0**
**<3.1**	82.7	66.2	53.4
**3.1-6.7**	79.3	66.4	54.5
**>6.7**	72.1	64.6	53.9

TIR, time in range; TBR, time below range; TAR, time above range; SD, standard deviation.

Supplementary Figure 1. Comparison of relative frequency of TIR between users of an integrated connected insulin pen and other LibreView users in Europe

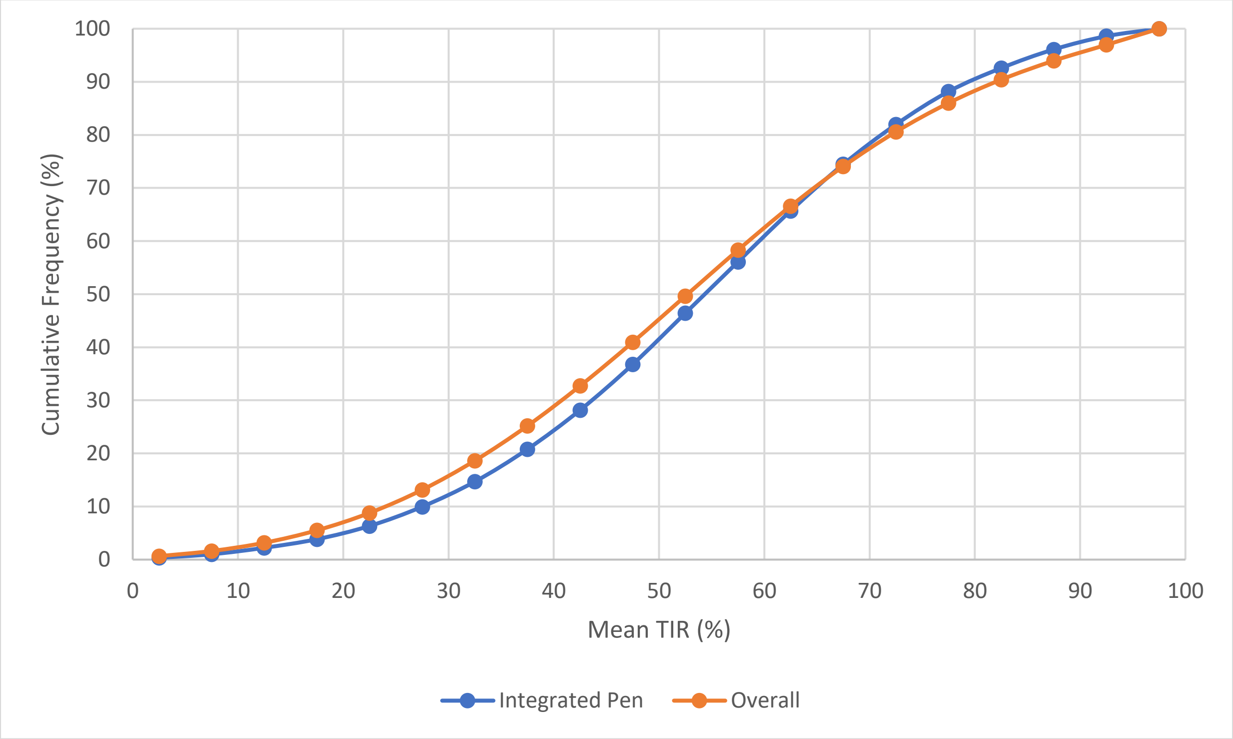

The figure shows that the TIR distribution of our analysis sample (blue) is similar to that of the overall LibreView database (orange) in the same time period (LibreView users with at least 30 days of readings following March 1, 2024 and no integrated connected insulin pen data; n: 669,305). The mean TIR of integrated pen users was 56.3%. Their 25th percentile, median, and 75th percentile of TIR were 42.9%, 56.8%, and 70.3%, respectively. The mean TIR of other LibreView users was 55.2%. Their 25th percentile, median, and 75th percentile of TIR were 39.9%, 55.2%, and 70.7%, respectively.

TIR, time in range
